# Event-Based Vision at the Edge: A Review

**DOI:** 10.3390/brainsci16040422

**Published:** 2026-04-17

**Authors:** Michael Middleton, Teymoor Ali, Epifanios Baikas, Hakan Kayan, Basabdatta Sen Bhattacharya, Elena Gheorghiu, Mark Vousden, Charith Perera, Oliver Rhodes, Martin A. Trefzer

**Affiliations:** 1 Department of Electronic Engineering, University of York, York YO10 5DD, UK; michael.middleton@york.ac.uk; 2 Faculty of Natural Sciences, University of Stirling, Stirling FK9 4LA, UK; t.r.ali@stir.ac.uk (T.A.); elena.gheorghiu@stir.ac.uk (E.G.); 3 School of Electronics and Computer Science, University of Southampton, Southampton SO17 1BJ, UK; e.baikas@soton.ac.uk (E.B.); m.vousden@soton.ac.uk (M.V.); 4 School of Computer Science and Informatics, Cardiff University, Cardiff CF10 3AT, UK; kayanh@cardiff.ac.uk (H.K.); pererac@cardiff.ac.uk (C.P.); 5 School of Electrical and Electronic Engineering, University of Manchester, Manchester M13 9PL, UK; basab@manchester.ac.uk (B.S.B.); oliver.rhodes@manchester.ac.uk (O.R.)

**Keywords:** neuromorphic computing, spiking neural networks, neuromorphic hardware

## Abstract

**Highlights:**

**What are the main findings?**

- There is poor integration between SNN architectures, training, datasets, and hardware, which limit deployment of event-based vision systems at the edge.
- Access to neuromorphic platforms is restricted by scarce, costly hardware (including compute substrates sensors) and related proprietary toolkits.

**What are the implications of the main findings?**

- Progress requires bridging gaps between research areas, rather than optimising each discrete area in isolation.
- Open datasets and synthetic data are expanding access to neuromorphic research despite hardware limitations.

**Abstract:**

Spiking Neural Networks (SNNs) executed on neuromorphic hardware promise energyefficient, low-latency inference well-suited to edge deployment in size, weight, and powerconstrained environments such as autonomous vehicles, wearable devices, and unmanned aerial platforms. However, a coherent research pathway to deployment of neuromorphic devices remains elusive. This paper presents a structured review and position on the state of SNN-based vision across four interconnected dimensions: network architectures, training methodologies, event-based datasets and simulation techniques, and neuromorphic computing hardware. We survey the evolution from shallow convolutional SNNs to spiking Transformers and hybrid designs which leverage the advantages of SNNs and conventional artificial neural networks. We also examine surrogate gradient training and ANN-to-SNN conversion approaches, catalogue real-world and simulated event-based datasets, and assess the landscape of neuromorphic platforms ranging from rigid mixed-signal architectures to fully-configurable digital systems. Our analysis reveals that while each area has matured considerably in isolation, critical integration challenges persist. In particular, event-based datasets remain scarce and lack standardisation, training methodologies introduce systematic gaps relative to deployment hardware, and access to neuromorphic platforms is restricted by proprietary toolchains and limited development kit availability. We conclude that bridging these integration gaps, rather than advancing individual components alone, represents the most important and least addressed work required to realise the potential of SNN-based vision at the edge.

## 1. Introduction

Artificial intelligence has transformed the landscape of embedded and edge computing, enabling capable perception and decision-making systems to operate in increasingly constrained environments. As these systems proliferate across domains such as autonomous vehicles, wearable devices, and unmanned aerial platforms, the computational demands of conventional deep learning approaches have become a central engineering concern. The dominant paradigm, built upon dense matrix operations and synchronous activation functions, was designed for data centres rather than for deployments where size, weight and power are the defining constraints. It is within this context that Spiking Neural Networks (SNNs) have attracted sustained research interest as a fundamentally different computational model, one that processes information through discrete spike events rather than continuous-valued activations, and that promises to deliver capable inference at a fraction of the energy cost of conventional artificial neural networks. The sparse, eventdriven nature of spiking computation means that processing occurs only in response to meaningful changes in the input, rather than continuously across all neurons at every timestep. When executed on neuromorphic hardware designed to exploit this sparsity, the resulting reductions in power consumption, silicon area, and system weight are potentially transformative for edge deployment. Neuromorphic platforms have demonstrated that these properties can be realised in hardware, while the emergence of event-based vision sensors has provided a sensing modality naturally aligned with spike-based processing.

However, despite considerable advances across hardware, algorithms, and datasets, a coherent and practical pathway from SNN research to deployed systems operating under real size, weight and power budgets remains a challenge. Training methodologies rely heavily on surrogate gradient techniques that approximate the non-differentiable spike function, creating a systematic gap between the model used during training and the hardware on which it is eventually deployed. Relative to existing approaches using frame-based sensors and Von Neumann processing architectures, event-based data collection and processing for computer vision is significantly more challenging. Evaluation practices remain inconsistent, with SNNs frequently benchmarked against conventional deep learning on datasets that do not reflect the conditions under which spiking computation offers its greatest advantages. Event-based vision data, the most natural input modality for SNNs, remains scarce and poorly standardised.

**Figure 1:**
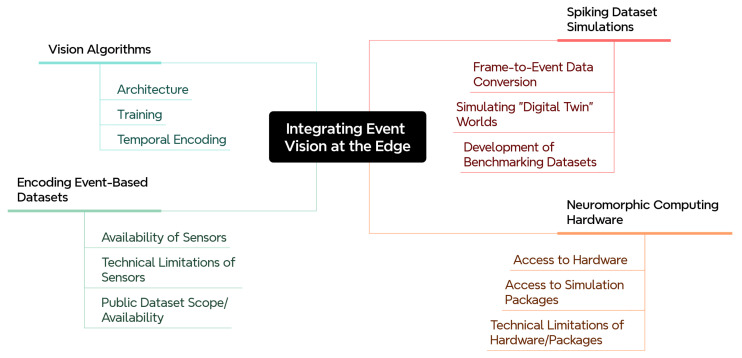
Overview of areas involved in deploying SNN vision algorithms at the edge, with associated gaps in integration that must be assessed.

This paper offers a structured account of the current state of SNN research through the lens of practical deployment and proposes a path to close the gap between theoretical promise and engineering reality. It argues that the field has made substantial progress in isolation across hardware, training, and sensing, but that bridging the integration challenges between these areas, and aligning them with the size, weight and power requirements of real systems, represents the most important and least addressed work ahead. By examining where these gaps are largest and what conditions would need to hold for SNNs to fulfil their promise at the edge, this paper aims to offer a clearer roadmap for the research directions most likely to matter. Figure 1 provides an overview of the SNN landscape structured around four key areas: network architectures, training methodologies, vision task applications, and current challenges. In what follows, we discuss each of these areas in detail.

## 2. Vision Algorithms

### 2.1. Network Architectures for Event-Based Vision

The landscape of SNN architectures for event-based vision has evolved considerably, from initial feedforward networks to complex deep architectures that rival their ANN counterparts in performance. Current state-of-the-art architectures can be broadly categorised into several groups that all differ in how information is routed and linked over space and time.

#### 2.1.1. Convolutional Spiking Neural Networks

**Figure 2:**
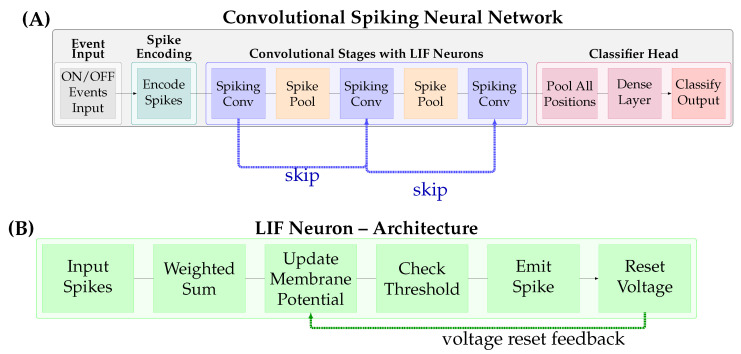
Generic Convolutional Spiking Neural Network (CSNN) for event-based vision. (**A**) Asynchronous ON/OFF events are encoded into spike trains and processed through three spiking convolutional stages each coupling spatial feature extraction with Leaky Integrate-and-Fire (LIF) neurons, interleaved with spike-preserving pooling. Dashed blue arcs below indicate residual skip connections. Features are aggregated globally and passed to a dense classifier. (**B**) Internal architecture of a single LIF neuron: input spikes are weighted and summed to update a membrane potential; once the threshold is crossed a binary spike is emitted and the voltage is reset via a feedback loop.

Convolutional Spiking Neural Networks (CSNNs), as illustrated in Figure 2, form a baseline architecture for event-based vision tasks, adapting the successful convolutional paradigm from traditional computer vision to the spiking domain.

As in ANNs, the CSNN topology is specified a priori: convolution enforces local receptive fields and weight sharing, yielding translation equivariance and a fixed interaction graph between neurons. As a result, information propagates primarily locally, with each neuron integrating spikes from a small spatial neighbourhood, whilst global structure (object-level context) emerges only after stacking multiple convolutional stages (and, in deep designs, residual pathways that ease gradient and signal propagation). Crucially, in CSNNs the temporal dimension is not introduced by dynamic routing, but by the neuron and synapse dynamics: incoming spikes are filtered and accumulated in membrane state variables over time, and outputs are emitted as discrete spikes when thresholds are crossed. Thus, spatiotemporal processing arises from combining (i) spatially local, translation equivariant convolutional connectivity with (ii) temporal integration and reset dynamics at the neuron level.

Operationally, each layer applies a shared kernel to local spike activity (often via synaptic filtering over a short time window), updates membrane potentials, and emits output spikes that become the input event stream for the next layer. Pooling then coarsens the spatial resolution whilst preserving the event-driven temporal representation, trading fine spatial detail for invariance and efficiency without changing the fact that temporal structure is carried in spike timing and state dynamics.

Modern CSNNs incorporate advanced features such as residual connections and normalisation techniques specifically adapted for the spiking domain [1,2,3]. Recent implementations have demonstrated that deep CSNNs can effectively extract hierarchical features from event streams, enabling robust performance in classification, detection, and tracking tasks [4,5,6]. The integration of these architectural innovations with spike-based computation allows CSNNs to leverage both the inductive biases of convolutional architectures and the temporal precision afforded by asynchronous event processing. This combination has proven particularly effective for high-speed vision tasks where traditional frame-based approaches struggle with motion blur and temporal aliasing.

#### 2.1.2. Spiking Transformer Architectures

**Figure 3:**
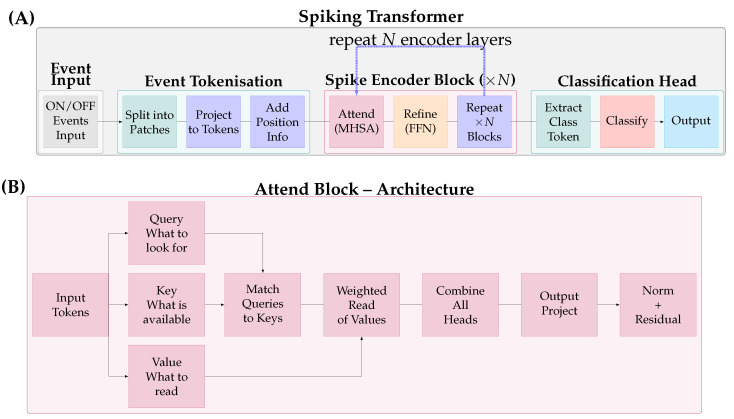
Generic Spiking Transformer for event-based vision. (**A**) Events are divided into patches, projected into spike token embeddings with positional information, and processed by *N* encoder blocks each comprising a Spike Multi-Head Self-Attention (MHSA) layer and a feedforward refinement step. The CLS token is extracted and classified. The dashed arc shows the *N*-layer repetition. (**B**) Internal architecture of the Attend (MHSA) block: spike tokens are projected into Queries, Keys, and Values; Query–Key matching produces a sparse attention map which selectively reads the Values; outputs from all heads are combined, projected, and residually normalised.

In contrast to CSNNs, spiking Transformer architectures (Figure 3) represent a recent breakthrough in scaling SNNs to larger, more complex tasks. These architectures integrate attention mechanisms into the spiking domain, enabling networks to selectively focus on salient features within event streams across multiple dimensions: spatial attention identifies relevant regions in the input field, temporal attention weights different time steps or event windows, and feature-based attention modulates informativeness across channels through multiplicative gating of spike trains. The challenge in developing spiking Transformers lies in adapting the continuous-valued attention mechanism to binary spike trains whilst maintaining the ability to capture long-range temporal dependencies and cross-channel correlations.

Recent implementations [6,7,8,9,10,11] have demonstrated competitive performance with ANN-based Transformers whilst maintaining the energy efficiency benefits of spike-based computation. These architectures typically employ spike-driven self-attention mechanisms where attention weights are computed from accumulated spike patterns, and query, key, and value transformations are adapted to operate on discrete spike representations. The multi-head attention mechanism enables parallel processing of different temporal scales, spatial regions, and feature abstractions, which is particularly valuable for processing the asynchronous event streams from event-driven cameras.

Despite these advances, spiking Transformers face limitations including the difficulty of accurately approximating softmax operations with discrete spikes [12], high memory demands from temporal unrolling, and performance gaps on large-scale complex tasks [13]. The current trends surrounding spiking Transformers is, at least in part, misplaced when the deployment context is the constrained edge. Transformers were conceived as scaling architectures, and their memory and compute footprint, even in spiking form, sits awkwardly with strict size, weight, and power budgets. Convolutional and recurrent SNN architectures, though less fashionable in the current literature, may ultimately prove better matched to the environments this field is most motivated to serve. Progress on spiking Transformers is valuable, but it should not crowd out architectural work on designs that are practical from the outset.

#### 2.1.3. Hybrid SNN-ANN Architectures

**Figure 4:**
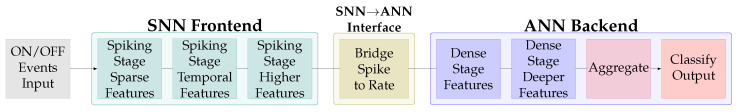
Cascade Hybrid SNN-ANN architecture. The SNN frontend (teal) processes asynchronous ON/OFF events through spiking stages, exploiting data sparsity for energy-efficient temporal feature extraction. A bridge layer normalises accumulated spike rates into continuous activations, connecting the discrete and analogue regimes. The ANN (blue) is trained to convergence using standard backpropagation.

Hybrid SNN-ANN architectures, depicted in Figure 4, address the performance gap between pure SNNs and ANNs by systematically combining their complementary strengths. Pure SNNs struggle with high-precision continuous outputs, whilst pure ANNs lack temporal processing advantages and energy efficiency of spike-based computation [14,15,16]. Hybrid strategies include cascade architectures that process data sequentially through SNN and ANN stages, parallel architectures that fuse outputs from both pathways, and task-specific designs that allocate operations based on computational characteristics, utilising SNNs for sparse event processing and ANNs for high precision outputs.

The systematic assignment of layers to either spiking or analogue processing enables optimised performance across the accuracy-efficiency spectrum. Early layers often utilise spiking operations to process sparse event inputs efficiently, whilst deeper layers transition to analogue computation for complex feature integration. The interface requires careful design to minimise information loss during spike-to-activation conversion. The proportion of SNN versus ANN layers directly impacts trade-offs between accuracy, energy consumption, and latency, with optimal configurations being task-dependent.

Training hybrid architectures presents unique challenges, as gradients must flow through both discrete spike-based and continuous activation layers. Progressive training approaches that convert and fine-tune layers sequentially have shown promise for maintaining gradient stability, whilst end-to-end methods must carefully handle gradient approximation at SNN-ANN interfaces to prevent error accumulation. Beyond training, hybrid architectures face limitations including empirical layer allocation lacking principled guidelines, interface complexity that can offset efficiency gains, and increased memory requirements [17]. Hybrid architectures, whilst useful, represent an engineering compromise rather than a principled solution. If the core motivation for SNNs is energy efficiency at the edge, then inserting ANN layers wherever SNNs underperform raises questions about what the approach is ultimately delivering. Hybrids may be best understood as a diagnostic tool: the layers that require ANN substitution reveal where spiking computation still has room to grow, and in that sense they are a useful map of open problems rather than an endpoint in themselves.

#### 2.1.4. Recurrent Spiking Neural Networks

**Figure 5:**
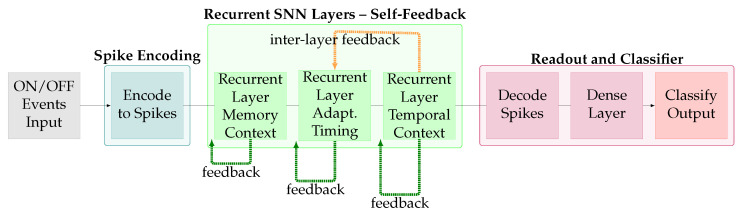
Generic Recurrent Spiking Neural Network (RSNN). Events are encoded into spike trains and processed through three recurrent spiking layers, each integrating both feedforward spikes and recurrent feedback from the previous timestep. Later layers use adaptive neurons with learnable time constants. Dashed green arcs show recurrent self-connections; the orange arc shows an optional inter-layer feedback path. Spike rates are decoded and passed to a dense classifier.

Recurrent Spiking Neural Networks, as shown in Figure 5, exploit the inherent temporal dynamics of spiking neurons to maintain and process sequential information through feedback loops, thus allowing the SNN to remember (but memory is distributed across space and time rather than stored). Unlike traditional recurrent architectures that require explicit recurrent connections, spiking neurons possess intrinsic memory through their membrane potential dynamics and leaky integration properties. This enables feedforward SNNs to extract temporal features without explicit recurrence, reducing parameter count and computational complexity.

Advanced recurrent SNN architectures incorporate mechanisms such as adaptive neuronal dynamics and learnable time constants, enabling networks to modulate their temporal processing characteristics based on task requirements [18,19]. The voltage leak and reset strategy in spiking neurones determines adaptation speed to new inputs, enabling networks to respond differentially to temporal order and timing variations in event sequences. Some architectures implement long short-term memory-style gating mechanisms adapted to the spiking domain, providing enhanced control over information flow and temporal context preservation.

#### 2.1.5. ANN to SNN Conversion

ANN-to-SNN conversion provides an alternative pathway that leverages wellestablished ANN training methodologies, thus preserving ANN performance while achieving the efficiency of SNNs. This approach involves first training a conventional deep neural network on the target task, then converting the trained parameters to an equivalent SNN representation. The conversion process maps ANN activation values to SNN firing rates, with the trained weights and biases transferred to the spiking network.

Modern conversion methods achieve remarkably low latency, requiring only four to eight timesteps to reach performance comparable to the source ANN, representing at least an order of magnitude improvement over early conversion techniques. The conversion typically involves rate coding, where the firing rate of spiking neurones approximates the activation values of the source ANN. Careful normalisation and threshold calibration ensure that firing rates remain within appropriate ranges across all layers.

However, conversion-based methods face inherent limitations. They cannot fully exploit the temporal dynamics and event-driven processing that define SNNs, and performance degrades when applied to tasks requiring precise temporal coding or processing of asynchronous event streams. The rate-coding assumption underlying most conversion methods is fundamentally at odds with the temporal precision available in event-based vision systems [20,21]. ANN-to-SNN conversion for edge deployment faces certain challenges. Networks produced by conversion inherit the dense, synchronous assumptions of their ANN source, which limits how well they can exploit the asynchronous, event-driven inputs that make neuromorphic hardware attractive. Conversion remains a useful research tool for understanding the relationship between ANN and SNN representations, but it may not be the most natural path to deployed neuromorphic systems.

Table 1 summarises recent state-of-the-art Spiking Neural Networks on ImageNet-1K, grouped by architecture and training paradigm: directly trained CNN- and Transformerbased models achieve competitive accuracy at only *T* = 4 timesteps, while ANN-to-SNN conversion methods reach higher peak accuracy at the cost of significantly more timesteps (*T* = 64), highlighting the fundamental trade-off between latency and performance in current SNN designs.

### 2.2. Vision Tasks

SNNs have demonstrated substantial capabilities across event-based vision tasks, with performance increasingly approaching or exceeding traditional deep learning methods in several domains.

#### 2.2.1. Object Detection and Recognition

Object detection and recognition constitute primary application areas where SNNs have shown considerable promise. Recent SNN based detectors achieve competitive mean average precision on automotive datasets whilst operating with significantly lower latency and energy consumption than conventional neural networks [32,33,34,35]. Event-guided object detection frameworks that fuse intensity images with event streams demonstrate that SNNs can effectively leverage both modalities, preserving texture details from conventional images whilst capturing high-frequency edge information from events.

Complex object detection architectures incorporate attention mechanisms that enable SNNs to selectively trigger at salient object locations, guided by event-based contour enhancement [36,37]. The binary-spike communication characteristics of SNNs are wellsuited to event-based vision, as they naturally suppress redundant computation in static or slowly changing regions of the visual field. Temporally integrating spikes enables the accumulation of evidence over time, improving detection confidence while maintaining responsiveness to visual changes.

#### 2.2.2. Optical Flow Estimation

Optical flow estimation naturally aligns with Spiking Neural Networks (SNNs) because motion is inherently temporal, and event cameras provide high temporal resolution with sparse, asynchronous measurements. In this setting, SNNs can exploit spike-based processing to reduce redundant computation and to better match the event-driven sensing modality. A key advantage of SNNs for event-based flow is their ability to maintain temporal context through membrane potential dynamics, which naturally integrate motion information over short time windows without requiring explicit frame boundaries. This supports low latency inference and efficient processing of continuous streams. In sparse optical flow formulations, computation is performed only at pixels where events occur, rather than densely across the full image plane, further improving efficiency when activity is localised [38,39,40].

Estimating dense optical flow from event data introduces additional challenges because the input is sparse and may not fully constrain motion in textureless regions or during low event rates. Hybrid formulations that combine events with intensity frames or aggregate events over time into pseudo frames can provide denser spatial support and enable the prediction of dense flow fields. Encoder–decoder architectures, including U-Net style designs with skip connections [41], are commonly used to fuse information across multiple spatial scales while preserving fine detail, and can be further extended with temporal processing to integrate event history when producing dense outputs.

#### 2.2.3. Action Recognition and Gesture Classification

Action recognition and gesture classification leverage the temporal processing capabilities of SNNs to extract motion patterns from event streams [42]. Research demonstrates that voltage integration in spiking neurones enables temporal feature extraction in feedforward networks without requiring explicit recurrent connections, offering parameter efficiency advantages over LSTM-based approaches [43]. SNNs achieve comparable accuracy to recurrent ANNs with significantly fewer parameters, demonstrating favourable trade-offs between accuracy and efficiency, particularly relevant for edge deployment.

The intrinsic temporal dynamics of spiking neurones, controlled by voltage-leak and reset strategies, determine adaptation speed to new inputs and enable networks to respond differentially to temporal-order and timing variations in action sequences. This proves crucial for distinguishing between actions with similar spatial characteristics but different temporal signatures. Event-based gesture recognition benefits particularly from the high temporal resolution of event cameras, enabling discrimination of fast hand movements that would appear blurred in frame-based capture.

Hierarchical temporal processing in deep SNNs enables the extraction of action primitives at lower layers and their composition into complex action representations at higher layers. Attention mechanisms guide the network to focus on relevant spatial regions and temporal segments, improving recognition accuracy whilst reducing computational requirements. The ability of SNNs to process continuous event streams without frame boundaries enables seamless recognition of actions with variable duration and complex temporal structure.

### 2.3. Discussion and Challenges

Despite significant progress, several fundamental challenges persist in advancing SNN-based event-based vision, spanning theoretical understanding, practical implementation, and community standardisation.

#### 2.3.1. Training Stability and Gradient Flow

Training stability and gradient flow remain a significant challenge in deep spiking networks. As network depth increases, spike vanishing becomes increasingly severe, with deeper layers receiving insufficient spike activity to generate meaningful gradients. The discrete binary nature of spikes, combined with complex spatiotemporal dynamics, can lead to unstable second-order moments in data streams, causing gradient explosion or vanishing. Recent work addresses these issues through adaptive normalisation techniques applied within spiking neurones, learnable neuronal dynamics that adjust temporal integration properties, and gradual surrogate gradient schedules that evolve during training [44,45,46]. Batch normalisation adapted to the spiking domain helps stabilise the distribution of membrane potentials across layers, preventing the accumulation of bias that can suppress spiking activity.

Initialising network parameters is vital for successful training. Poor initialisation can lead to dead neurones that never spike or saturated neurones that spike excessively, both of which prevent effective learning [47]. Specialised initialisation schemes that account for the spiking activation function and temporal dynamics help establish appropriate initial firing rates across layers [48]. Weight initialisation strategies must account for the temporal accumulation of membrane potentials and the resulting firing patterns to ensure stable gradient flow from the outset of training [49].

Gradient flow challenges become particularly acute in networks with recurrent connections or long temporal dependencies. The interaction between spatial and temporal depth leads to compound gradient attenuation, requiring gradients to propagate backwards through network layers and through time [50]. This dual propagation requirement necessitates careful architectural design and training protocols to maintain effective learning signals throughout the network.

#### 2.3.2. Latency and Timestep Requirements

Latency and timestep requirements present a trade-off between accuracy and responsiveness in SNN deployment [51]. Whilst converted SNNs achieve reasonable performance with four to eight timesteps, directly trained SNNs optimised for temporal tasks may require longer simulation windows to accumulate sufficient spike information and stabilise recurrent dynamics. The warm-up period required for hidden states to stabilise before meaningful computation occurs introduces additional latency that can be problematic for real-time applications.

The number of timesteps directly impacts both computational cost and energy consumption, as each timestep requires processing events across all active neurons and synapses. Reducing timestep requirements whilst maintaining accuracy remains an active research direction, with approaches exploring architectural innovations, improved encoding schemes, and specialised training techniques [52,53,54]. Learnable time constants represent one promising direction, enabling networks to automatically adjust temporal integration properties during training to find optimal trade-offs between temporal precision and computational efficiency. These adaptive mechanisms may reduce the number of timesteps needed for convergence by allowing different layers or neuron populations to operate at different temporal scales appropriate to their computational function.

Burst coding [55] and population coding [56,57] schemes offer alternative approaches to conveying more information per timestep, potentially reducing latency requirements. However, training networks with these complex temporal codes introduces additional demands, requiring specialised learning rules and careful architectural design to effectively exploit the enhanced representational capacity.

#### 2.3.3. Benchmarking and Evaluation

The SNN research area faces significant challenges in establishing standardised benchmarking protocols and evaluation metrics. Unlike traditional deep learning, where frameworks such as ImageNet classification and COCO object detection provide common reference points, event-based vision lacks comparable standardisation. This absence hinders objective comparison of different approaches and impedes assessment of progress in the field. However, efforts are underway to establish unified evaluation frameworks and shared datasets [58,59,60,61].

Evaluation metric inconsistencies further complicate comparison across studies. Different research efforts employ varying temporal window lengths, ranging from tens to hundreds of milliseconds, making direct performance comparisons difficult. Energy consumption measurements lack standardisation, with some studies counting only spike operations, whilst others include membrane potential updates, and few accounting for hardware-specific energy costs that vary substantially across platforms [62]. Temporal encoding variations introduce additional evaluation challenges. Studies employ different encoding schemes, including rate coding, temporal coding, and hybrid approaches, each with distinct computational characteristics and performance implications [63]. The lack of consensus on optimal encoding strategies across task categories hinders meaningful comparisons and prevents the consolidation of standardised best practices.

## 3. Dynamic Vision Sensors and Encoding Spiking Data

Conventional image sensors, such as frame-based cameras, capture data synchronously across all pixels in the sensing array. During each exposure interval, the photo sensor integrates incident light acting on the sensor and then transmits the intensity information for every pixel simultaneously as a complete image frame. As a result, the output bandwidth of frame-based sensors is high as information is captured and transmitted synchronously for all pixels in the scene, even if the information at a pixel remains unchanged from the previous frame. Furthermore, the typical rate of capture for framebased sensors is 20–30 ms per frame. If a feature in the captured scene moves a significant distance as the frame is being captured, it will appear “blurred” from its motion in the output data stream.

In contrast, a Dynamic Vision Sensor (DVS), also known as an event-based camera, operates asynchronously at the pixel level. Each pixel in a DVS independently detects and reports changes in brightness, generating an event only when the change exceeds a predefined threshold. Consequently, when the visual scene is static, the DVS produces little to no output data, resulting in lower redundancy and more efficient information encoding [64]. Unlike frame-based sensors, the rate of capture of pixels in the DVS array is measured in microseconds, which mitigates against motion blur in data.

Dynamic Vision Sensors belong to a broader class of neuromorphic and bio-inspired sensing technologies that emulate the signal processing strategies of biological vision systems. In biological retinas, photoreceptor and ganglion cells operate asynchronously, transmitting information primarily in response to changes in luminance rather than absolute intensity levels. This event-driven encoding allows biological systems to process visual information with remarkable efficiency, high temporal precision, and minimal redundancy [64].

**Figure 6:**
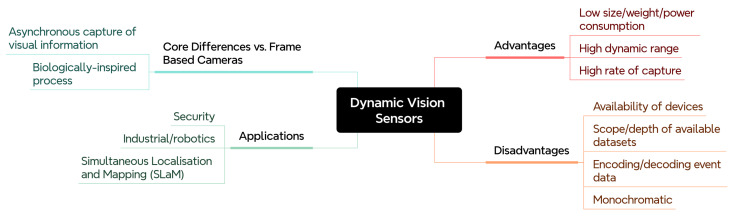
An overview of the motivations, applications and present-day challenges researchers may encounter when working with dynamic vision sensors.

Dynamic Vision Sensors adopt similar principles by integrating sensing and processing elements that respond to temporal changes in the input signal. In this section, a review of popular and emerging event-based vision datasets for neuromorphic learning are presented, followed by a discussion of the challenges learning from event-based vision data. An overview of the topics explored here is provided in Figure 6. Additionally, reviews have been conducted on dynamic vision sensors [65] and event-based vision more generally [66].

### 3.1. Address Event Representation

Event-based vision data is represented as continuous streams of discrete events, rather than as a sequence of image frames. Each event corresponds to a localised change in brightness detected at a specific pixel within the sensor array. The resulting data can be interpreted as a spatio-temporal sequence of brightness changes, or equivalently, as asynchronous spike trains distributed across the pixel array.

An event typically encodes the pixel’s spatial coordinates, the precise timestamp of occurrence, and the polarity of the brightness change, indicating whether the local luminance increased or decreased. This is known as Address-Event Representation (AER). In practice, manufacturer-specific implementations of AER are often used to encode event data from the DVS. There are currently no industrial standards defining AER, although the polarity, timestamp and X/Y position components are common between all formats of AER. Additional information can also be sent and received from some models of DVS, such as external trigger signals for synchronisation.

Manufacturers producing event-based vision sensors include Prophesee, iniVation, Sony and H.P.B. Optoelectronics. Currently, Prophesee and iniVation are the leading DVS manufacturers, with both taking a different approach to AER encoding.

#### 3.1.1. EVT (Prophesee)

Event Type (EVT) encoding is used by Prophesee for their range of dynamic vision sensors. The EVT 2.0 format is used with the IMX636 and GenX320 event-based sensors, and encodes “on” (brightness increase) and “off” (decrease) events as 32-bit packets [67]. Two packets are transmitted to describe one event, for a combined total of 64 bits of information. 11 bits are allocated for both the X and Y coordinate, allowing for a maximum spatial resolution of 2048 × 2048 to be represented in this format. The timestamp is encoded as 32 bits and polarity is encoded as a single bit. The remaining bits are unused as part of the AER representation. EVT 3.0 is the latest version of the format [68]. Here, event data is transmitted as 16-bit packets to improve data compactness. The structure of the AER is more complex, as sensors using EVT 3.0 achieve data compactness by encoding and vectorizing the sensor data. This requires the DVS to manage an internal state representation.

#### 3.1.2. AEDAT (iniVation)

An alternative AER format is the Address Event Data (AEDAT) format, used by iniVation dynamic vision sensors. AEDAT 1.0, used with the DVS128 sensor, encodes AER data in 6 byte-long strings, where the timestamp occupies 4 bytes (32 bits) [69]. The remaining 2 bytes (16 bits) encode the X and Y coordinate of the event using 7 bits each, and polarity is encoded as a single byte. The AEDAT 1.0 format therefore has a maximum spatial resolution of 128 × 128. Given this limitation on resolution, the AEDAT 1.0 format has been depreciated.

AEDAT 4.0 is the most recent iteration of the format, released in 2019 [70]. It is a container format for event-camera data built using Google FlatBuffers [71]. Instead of a raw stream of data, events are organised by type into packets. Packets can describe brightness changes, but many other events are supported depending on the model of sensor used (such as events describing an external input trigger, or if frame-based data has been captured). Packets are then grouped into streams with their own metadata and 64-bit timestamps in Unix time format, optionally LZ4-compressed to lower the memory overhead of data transmission.

### 3.2. Real-World Event-Based Vision Datasets

Similarly to benchmarking datasets in conventional computer vision (such as the MNIST family), there have been datasets proposed for Spiking Neural Networks that are designed for the evaluation of SNNs in a research environment. Some datasets extend beyond abstract benchmarking tasks and address real-world applications, such as the autonomous driving of vehicles. In the following sections, several popular event-based vision datasets are discussed and their application towards different applications of computer vision are examined. The datasets examined are summarised in Table 2.

#### 3.2.1. N-MNIST and MNIST-DVS

In image recognition tasks, the MNIST handwritten digit dataset [78] has been used to benchmark neural network performance in many studies [79,80,81,82,83]. MNIST is a classic benchmark dataset of 70,000 greyscale images of handwritten digits (0–9), each sized 28 × 28 pixels, and split into 60,000 training images and 10,000 test images.

An event-based representation of the MNIST dataset, known as N-MNIST dataset, has also been produced as a derivative from MNIST [72]. To produce each example in the N-MNIST dataset, a DVS was attached to a surface which performs controlled microsaccadic motions, changing the focal point of the camera over time. The result is a dataset of event-based recordings for the same 70,000 digits, commonly used to benchmark Spiking Neural Networks and neuromorphic vision algorithms.

The N-MNIST dataset has been used in the benchmarking of SNN architectures to evaluate the class separation abilities of different configurations [84]. As part of this study, it was discovered that the N-MNIST dataset does not encode temporal information as the timings of spikes are not important to the SNN when learning a solution via bio-inspired plasticity rules. Therefore, the N-MNIST set encodes spatial information only, and is not suitable when the performance of spatio-temporal classification by an SNN must be analysed.

An alternative event-based representation of the MNIST set, known as the MNIST-DVS dataset has also been produced [73]. Temporal data is encoded by displaying a digit from the MNIST set on a LCD monitor, then slowly moving the digit on the screen rather than the vision sensor as in the N-MNIST approach. Meaningful spatio-temporal data is captured by the DVS as the relationships between spike timings describe the motion vector (direction and magnitude of translation) of the digit on the screen. This is opposed to the movement of the camera over the digit in the case of N-MNIST, where the temporal component does not describe any additional information about the scene [84]. The spiking events in an example from the N-MNIST set could be summed to recover an approximate description of the spatial content in the example, whereas summing the events from a MNIST-DVS example results in the spatio-temporal structure of the data being destroyed.

#### 3.2.2. N-Caltech101

The Caltech101 contains photographs of single everyday objects as the focus of the image, plus some background content containing less relevant features. It contains 101 distinct classes of objects, with each class containing between 40 to 800 examples of each [85]. The resolution of all examples are roughly 300 × 200 pixels, as samples have variable resolution. Like the MNIST handwritten dataset, it has been used to evaluate the performance of deep artificial neural networks [86,87,88,89,90].

The N-Caltech101 dataset is produced in a similar manner to the N-MNIST set; by attaching a DVS to a moving surface and generating the event data by emulating saccadic motion, and pointing it towards a monitor displaying samples from the dataset [72]. Examples from the Caltech101 dataset are displayed on a screen for capture by the DVS. Therefore, the N-Caltech101 dataset also only encodes spatial information, and the timings between spikes in the dataset are not important towards the recovery of features from the data.

#### 3.2.3. N-CARS

**Figure 7:**
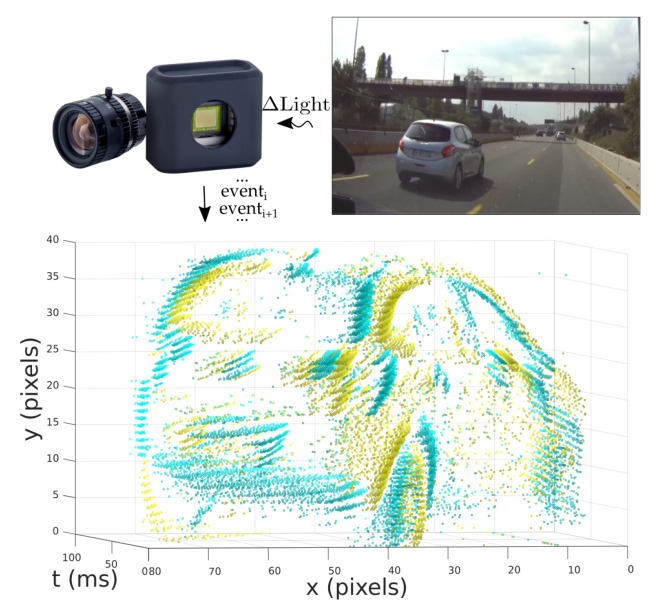
A representation of how data is recorded in the N-CARS dataset. The DVS mounted in the car captures light from the driver’s point of view, and outputs a stream of events. Figure adapted from [72].

The N-CARS neuromorphic vision dataset is an event-based vision dataset for binary classification [74]. The content is captured using an ATIS DVS mounted behind the windscreen in a moving vehicle. The two classes defined are “car” and “non-car” (or “background”). The “car” class describes data containing another car as seen from the perspective of the driver of the vehicle. Accordingly, the “non-car” class describes data where no other cars are in the scene. An example of event data representation in the N-CARS dataset is shown in Figure 7.

The dataset contains approximately 12,000 “car” and “non-car” samples, with each spatiotemporal sample capturing 100 ms of activity. The relatively short length of the temporal windows used for each example makes N-CARS an appealing dataset to study when the training of a neural network from highly sparse data is concerned.

Naturally, N-CARS is a dataset of interest in the field of autonomous driving, as determining if a road is occupied by another vehicle or not is a fundamental issue to be addressed. In a study by Viale et al. [91], a neuromorphic binary classification network was developed and hosted on neuromorphic hardware (an Intel Loihi processor). As the network is trained through spatio-temporal backpropagation [48], which is incompatible with the Loihi chip, it is trained “offline” on conventional Von Neumann architecture then hosted on neuromorphic hardware. At a spatial input resolution of 100 × 100 pixels, the CarSNN developed as part of the study achieved 86.3% accuracy in “offline” tests and similar performance in “online” tests. Furthermore, the energy used by the Loihi chip hosting the CarSNN architecture is low, at 319.7 µs per inference. A later study by Bano et al. investigated the role of parameter tuning in processing the N-CARS data for classification by an SNN, and improved training times by approximately 90% above the contemporary state-of-the-art performance [92]. A further study by Loïc et al. [93] investigated the role of SNN architecture on N-CARS classification. Their work identified the VGG-11 SNN architecture as being the highest-performing design, achieving 92.4% accuracy.

#### 3.2.4. MVSEC

The Multi-Vehicle Stereo Event Camera (MVSEC) dataset is a more complex dataset targeted towards automated driving applications [75]. As implied by its name, sensors were mounted to a variety of vehicles including drones (hexacopters) and motorbikes. The dynamic vision sensors are positioned in a way to capture stereo vision in a bioinspired manner, allowing for the perception and calculation of depth from the difference in stereo vision. Additional sensors also record LiDAR, GPS and motion capture information. See Figure 3 from Zhu et al. [75] for a visualisation of frame and event data from the MVSEC dataset.

MVSEC can therefore be of interest beyond automated driving applications and be used to examine general stereoscopic vision processing principles in SNNs. This is demonstrated in a study by Rançon et al. [94]. A spiking architecture similar in structure to a U-net was trained on the stereoscopic dataset to model scene depth at the pixel level using surrogate gradient descent. Experiments by Kosta and Roy demonstrated a fully-spiking architecture (“Adaptive-SpikeNet”) modelling optical flow [95]. Whilst the overall accuracy of the spiking networks investigated was lower than non-spiking architectures, the spiking network used almost half as many parameters and 10% of the energy. Similarly, research by Lee et al. demonstrates a hybrid SNN-ANN approach to estimating optical flow from the MVSEC dataset [96]. Their Spike-FlowNet architecture also adopts a U-net-style structure, featuring spiking encoding blocks and ANN residual and decoding blocks to construct optical flow from accumulated spikes. Compared to a fully ANN architecture, the spiking encoder block reduces the number of computations required by the network by 17.6%, implying a significant associated reduction in power consumption.

#### 3.2.5. DVSGesture

The DVSGesture dataset contains 11 classes of gestures performed using the hands and arms of subjects in an otherwise static scene [76]. Therefore, unlike N-CARS, it is suited towards the investigation of multi-class classification problems using SNNs. Despite the background content of the scene being static, there is some background noise captured by the DVS sensor and represented in the dataset due to minute changes in brightness or the motion of clothing. This demonstrates the sensitivity of dynamic vision sensors to small movements in the scene, and presents a challenge towards the recognition of gestures by an artificial neural network; the relevant features must be separated from the irrelevant and noisy features.

A pre-processing method for the denoising of DVSGesture data has been proposed by Zhang et al. [97]. They identify several sources of noise, including “hot pixel” noise caused by the circuitry of the DVS and background activity noise. The processing methods developed to remove these noise sources improved spatio-temporal correlation between source and target feature spaces and up to a 10% improvement in prediction accuracy. This demonstrates that the noise captured by event-based sensors can significantly affect the prediction accuracy of artificial neural networks, especially when there is correlation between sources of noise between samples of different classes (such as a consistent background causing semi-regular patterns of noise to emerge around non-relevant background features).

A later study by Rizzo et al. [98] used a fully-neuromorphic method of spatial reduction which, given its functionality, implicitly filters some sources of noise from the DVSGesture data. Their work uses a spatio-temporal collapsing scheme to represent highlyactive areas of the source data as “chips” with a state of 1. Accordingly, less active areas of the spatial domain are represented with a state of 0. This approach pools spiking activity into a spatio-temporal representation with coarser sampling in space and time. Assuming that the signal-to-noise ratio of the event data is sufficient, this windowing approach removes noise by the filtering of less active spikes from the data.

#### 3.2.6. E-VLC

The Event-based Visible Light Communication and Localisation (E-VLC) is a recently developed dataset that the positions of objects in a scene by capturing the emission of light from blinking LEDs [77]. The dataset consists of events recorded by a Prophesee IMX636 1280 × 720 event sensor and frames recorded by a Basler acA1300-200 μm 1280 × 1024, both mounted side-by-side on a flat board. The capture of events and frames are synchronised using a trigger box mounted to the front of the camera setup that sends a pulse signal at 120 Hz. E-VLC is notable for its high resolution spatio-temporal representation of both event and frame data in comparison to other datasets. This is in comparison to other available datasets that have became established in the literature, such as DVSGesture (spatial resolution of 128 × 128) and N-MNIST (34 × 34).

**Figure 8:**
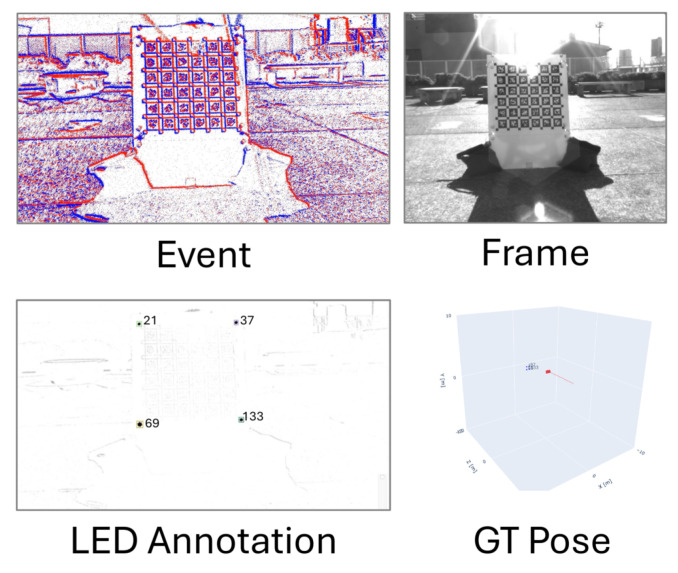
An example of the event and frame data captured by the handheld recording device used to produce the E-VLC dataset. The positions of LEDs fixed to the object and the position of the capture device relative to the object are also shown. Reproduced with permission from Shiba et al., “E-VLC: A Real-World Dataset for Event-based Visible Light Communication And Localization” [77]; published by the IEEE/CVF Conference on Computer Vision and Pattern Recognition (CVPR) Workshops, 2025.

A scene captured by these sensors consists of a small object, such as a box or table, with blinking LEDs fixed to it. This allows for the estimation of object location in 3D space and the movement of the object or capture device. Labels are generated by feeding the camera input into OptiTrak motion capture software, which provides a ground-truth estimation of position and movement by tracking reflective markers fixed to the object. The position of the camera itself is also estimated as part of the labelling process, recalculated 100 times per second at sub-millimetre spatial accuracy. The dataset does not describe the shape of the object for identification, only its position in a volume of space. An example of the frame and event data captured, along with the associated labelling data, is shown in Figure 8.

Multiple scenes are captured, including outdoor and indoor environments at different levels of illumination. For each of these scenes, multiple scenarios are captured. Over 20 examples are captured for each luminance level in a static scenario, where the capture device remains motionless for the duration of the recording. The camera can also experience movement through rotation or translation at walking speed. Between two and six examples are captured at each luminance level under these conditions. Finally, dynamic scenes feature 35 markers attached to multiple objects in the scene. The capture device passes through the scene, replicating the point of view of a person walking through the array of objects. Between three and five examples are recorded for dynamic environments.

As the E-VLC has been publicly available since 2025, there are no external studies using it to train a Spiking Neural Network model. Given the simultaneous capture of events and frames, the dataset may be of interest to studies investigating hybrid frame and event-based modelling. However, the low number of examples captured for translation, rotation and dynamic environments could limit the usefulness of the dataset in these scenarios.

### 3.3. Discussion and Challenges

The fundamental issue in working with event data from a dynamic vision sensor is the access to data. Researchers are limited first by access to an event-based camera, which are far less available than their frame-based alternatives. Secondly, accessing the data captured by the DVS often requires proprietary software from the manufacturer. Although the research community has enforced AER as a standard approach to representing the event data produced by a DVS, the lack of proper industrial standardisation makes recording and decoding the event streams from different manufacturers non-trivial in practice.

Event-based image sensing is still an emerging technology; for practical real-world applications presently, it is simpler to deploy a system using a frame-based camera. As discussed, using a frame-based sensor imposes restrictions on the size, weight and power consumption of a device deployed at the edge due to the synchronous nature of processing. From this review, it is anticipated that new possibilities in computer vision will emerge using dynamic vision sensors in scenarios at the edge which are currently restricted by the size and power consumption of frame based devices as the research supporting event-vision technology continues to mature.

Presently however, the limited availability of event-based sensors relative to their frame-based counterparts presents a significant challenge to researchers of neuromorphic vision tasks. The datasets presented in this section are applicable to a range of real-world scenarios, such as object and feature recognition, or spatial localisation. The complexity of datasets proposed by researchers is also diverse; some (such as N-MNIST) are relatively simple, low resolution and encode only spatial information. Others, such as MVSEC and E-VLC, consist of multiple sources of data, such as GPS position and synchronous frames.

In summary, the limited availability of hardware often restricts the scope of neuromorphic vision tasks to what is represented in publicly available datasets, making the integration of a neuromorphic vision system into a pipeline more difficult than a conventional frame-based camera. There is also a need to standardise the format of event data to simplify the access to data from dynamic vision sensors. These factors make the integration of dynamic vision sensors into an event-based computer vision system challenging at the present. Through improved access to dynamic vision sensors by some means, and by streamlining the access to data from these sensors, these integration challenges can be addressed.

## 4. Simulation of Spiking Datasets for Computer Vision

**Figure 9:**
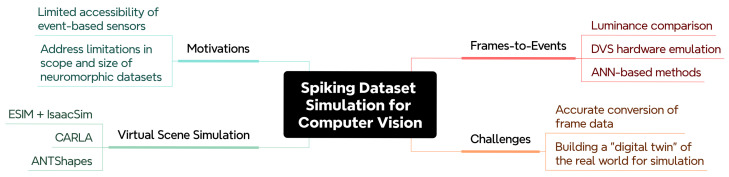
An overview of the motivations, approaches and challenges faced when simulating event data computationally.

The relative lack of event-based vision data to train Spiking Neural Networks is an obstacle that must be addressed to research and develop effective systems. To address this limitation, methods to simulate spiking data from sequential frames have been proposed, using the availability of frame-based vision data as leverage. Alternatively, some methods of simulating visual event data favour an entirely synthetic approach. In a fully-synthetic spike generation pipeline, a scene is rendered virtually in a 3D engine (such as Blender, Unity or Unreal Engine) and spiking data is estimated from information captured by a virtual camera in the scene. Both of these methods are explored in this section towards the simulation of event data for computer vision. An overview of the topics explored is presented in Figure 9, and the software tools discussed is given in Table 3.

### 4.1. Spike Estimation from Luminance

Recall from Section 3 that a dynamic vision sensor emits events when a change in brightness exceeds a threshold in the positive or negative directions. Following this principle, software solutions can be developed to compare the per-pixel luminance between frames to generate spiking data. Practically, this allows for existing datasets captured by frame-based sensors to be represented in an AER format similar to the output data stream from a DVS. As the number of neuromorphic vision datasets captured by event-based sensors available to researchers is relatively low compared to the number of frame-based video datasets, this approach can mitigate against limitations in data availability. Rather than detail existing datasets generated using this approach, this section will cover existing methods and software of converting frame information to spikes.

The spike estimation of a DVS is emulated in software by comparing the per-pixel difference in brightness to a threshold value. Image sequences captured at typical frame-rates (i.e., 24–60 frames per second) may not yield usable spiking information after conversion if motion blur dominates in the scene. Furthermore, the low rate of capture results in a high number of spiking events being generated between frames. This is due to the relatively long window of opportunity for the movement of features to occur between frames, potentially causing a large difference in brightness between frames.

Spiking events can then be extracted from the video footage by comparing the brightness between subsequent at a pixel level and evaluating the difference against a threshold value [107]. If the value is above the threshold, an event with positive polarity is generated signifying an increase in brightness. Conversely, if the difference is below the sign of the threshold, an event with negative polarity is generated indicating that the brightness at that pixel has decreased. Otherwise, no events are recorded, simulating the sparse representation of scenes captured by event sensors. Changing the value of the threshold alters the simulated dynamic range of the camera and can be used to adapt between lighting conditions between different scenes, similar to the behaviour exhibited by biological vision sensors.

The method presented by Kaiser et al. [107] is only applied to low temporal resolution video footage. Therefore, the low-latency behaviour displayed by event-based cameras is not replicated. To address this limitation, an open-source event simulator, ESIM [99], has been proposed to extract event data from image sequences by first interpolating between the source video frames using an temporal sampling technique that is adaptive to the amount of motion present in the scene. This approach satisfies the low-latency behaviour of a DVS by artificially simulating a high rate of capture by the frame-based sensor.

### 4.2. Emulation of DVS Circuitry

Another approach to simulating event data from frame sequences is by modelling the DVS circuit in software. This method seeks to emulate the method of event generation from luminance directly, including noise from sources within the circuitry, by building computational models of these noise sources. The difference in luminance intensity between frames is still computed, and the sampling scheme between frames is designed to replicate the emission of events by a DVS comparator circuit.

#### 4.2.1. DVS-Voltmeter

DVS-Voltmeter introduces noise to the frame-to-event model in several phases [100]. Firstly, noise from the arrival of photons to the receptor are modelled by applying Brownian motion to the timestamp to simulated events, offsetting the time of arrival. This source of noise is introduced to brightness-dependent randomness in the counting of photons by the photoreceptor circuit. The effect of this noise causes the change in voltage to become non-linear as drift is introduced to event timings. Noise caused by parasitic currents also affects the change in voltage sensed by the DVS. It is also modelled by Brownian motion in DVS-Voltmeter, as Brownian motion is the temporal integral of white noise, which is assumed to represent the circuit noise in the DVS. The noise model introduced by DVS-Voltmeter is therefore a statistical model of noise measured from real-world sensors.

#### 4.2.2. Raw2Event

Raw2Event is a recently proposed derivative of the DVS-Voltmeter method [101]. Rather than processing RGB-format frames, Raw2Event estimates events from raw data captured by the frame-based sensor. Camera hardware settings such as auto focus and exposure are disabled, and a Raspberry Pi single-board computer is used to read single 10-bit Bayer frames directly from the sensor. This bypasses software image processing in the camera, such as tonemapping and autogain, preserving per-pixel brightness. The DVS-Voltmeter approach is then applied to the raw frames, which can operate in real-time or “offline” on pre-prepared raw frames. Raw2Event is notable for being the first method proposed of synchronous raw frame and event generation at real time using conventional frame-based hardware.

The authors note that the devise methodology is implemented on readily-available hardware and can address the issue of the low availability of event-based sensors [101]. Furthermore, Raw2Event can address the limitations on resolution imposed by event-based sensors. To the author’s knowledge, the highest-resolution event-based sensor that has been developed measures 1280 × 960 pixels [108]. Frame-based sensors can capture at much higher resolutions. Therefore, a pipeline featuring a high-resolution frame-based sensor paired with event generation using Raw2Event could be used for applications where spatial and temporal resolution are critical. However, the Raw2Event pipeline may not be suitable for applications at the edge where low-power considerations are critical, as true dynamic vision sensors will consume less power given their sparse mode of capture and signal transmission.

### 4.3. ANN-Based Methods

The methods of frame-to-spike conversion discussed so far are algorithmic approaches towards estimating the spiking activity between frames captured at a synchronous rate, achieved by sampling interpolated luminance intensity between frames at a per-pixel level and applying the fundamental logic behind event generation using a DVS. The following approaches use ANNs to directly estimate events from a frame-based input.

#### 4.3.1. EventGAN

EventGAN uses a Generative Adversarial Network (GAN) to estimate events from frame data [102]. The method estimates a 3D spatiotemporal volume of events from a pair of simultaneous frames. The network architecture is similar in design to a U-net. The adversarial method of training the network constrains it to model realistic DVS behaviour, including sensor noise. Self-supervision is applied by feeding the predicted event volumes into a pair of pre-trained ANNs, which construct representations of optical flow and frame data from predicted events. Additional loss terms are then generated based on the difference between predicted and true frame and optical flow data, and are used as additional sources to train the GAN.

EventGAN predicts event data directly from pairs of frames. Alternatively, ANN-based methods may be used to interpolate between frames, before methods of estimating events from the interpolated frame data are applied. A study utilising the Super SloMo [109] method of frame interpolation to allow for event reconstruction at arbitrary time resolutions has been proposed by Jiang et al., using ESIM is used as a rendering backend. The Super SloMo method uses bi-directional ANN-based optical flow estimations to adaptively generate interpolated frames between source images, such that the content of any pixel in the array moves no further than 1 pixel in any direction in the frame period. The method was evaluated on the N-Caltech101 dataset by comparing simulated spikes extrapolated from downsampled footage to ground-truth spikes from the DVS. It was demonstrated that the Super SloMo interpolation method was suitable for reconstructing the spiking output from a DVS given the strong correlation between the pairs of ground-truth and interpolated data.

#### 4.3.2. Super SloMo Approaches

A Python toolbox to convert frame data to events using the Super SloMo interpolation method, known as the Video to Events (v2e), has also been proposed [103]. v2e introduces some sources of noise to mimic the non-ideal characteristics of an event-based sensor by allowing the threshold of the event detection mechanism to fluctuate, amongst others. In other words, the luminance threshold of each pixel over time is measured as a distribution rather than a constant value. This is in contrast to ESIM which does not introduce noise sources to its event estimation pipeline. Therefore, ESIM assumes non-physical ideal conditions as part of its frame-to-event processing pipeline.

The Video to Continuous Events (v2ce) simulator uses sequences of 16 frames to estimate events rather than pairs of frames as v2e does [104]. This allows for the prediction of events from non-linear changes in luminance intensity at the pixel level, whereas the prediction of events from pairs of frames implies a linear change in luminance between the two time steps. The predictions from the U-net-style ANN in the v2ce pipeline is a 3D spatiotemporal grid of cells, where each cell describes a map of the estimated cell density at each discrete (*t*, *x*, *y*) position. Events are then estimated from this density map, allowing the data to be read in an AER-style format. Furthermore, v2ce introduced quantifiable metrics towards the benchmarking of frame-to-event data conversion, and found that v2ce outperformed ESIM, EventGAN and v2e.

### 4.4. Fully Synthetic Data Simulation

As opposed to capturing information from the real-world using a vision sensor (either event or frame-based), event data can be simulated entirely computationally. A distinction is made here between the synthetic generation of events from real-world data captured by a frame-based camera, as discussed in the previous section, and entirely synthetic data captured inside of a virtual scene modelled in a 3D rendering engine such as Blender, Unity or Unreal Engine. This entirely synthetic approach is beneficial when a scene with a specific set of conditions is required for study which is not represented in a real-world dataset available to the researcher.

In Section 3.2.6, it was noted that the usefulness of the E-VLC dataset could be limited when studying non-static environments given the low number of examples represented for these scenarios. A reason for the low number of examples could be due to the physical cost of recording visual information in the real world for the dataset; objects had to be fitted with LEDs for the task, then placed in an environment large enough for the motion to be recorded, lighting levels set, and so on. Conversely, visual data could be simulated entirely computationally to represent the same scenario, removing the real-world limitations imposed on the dataset construction process.

Furthermore, the content in virtual scenes can be controlled to limit sources of noise that are present in real-world datasets. As discussed in Section 3.2.5, noise can adversely affect the ability of a neural network to learn from its data. Filtering the noise from the training data can be useful here, but preventing the noise from existing in the data to begin with can be a more effective approach as there is no opportunity to destroy relevant data. Therefore, simulating “ideal conditions” for training by limiting the simulated content to only the features of interest can also be used as a method of validating the SNN methodology before applying the approach to real-world datasets.

#### 4.4.1. ESIM and IsaacSim

Beyond frame-to-event data conversion, ESIM is designed to model the functions of an event-based sensor faithfully to the real-world [99]. As such, ESIM utilises a 3D engine to simulate and render scenes created using other 3D software, such as Blender and Unity. Therefore, a scenario can be created and scripted in an external program, then loaded into ESIM for event capture that accurately replicates dynamic vision sensor behaviour.

**Figure 10:**
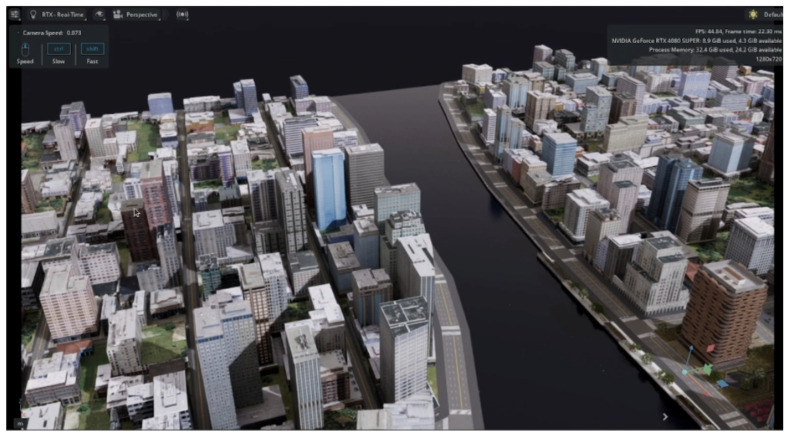
A virtual environment, representing a cityscape with densely-packed buildings, as rendered in IsaacSim. The simulator is capable of rendering realistic scenes and scripted behaviour expressed by entities within them.

NVIDIA IsaacSim is such a 3D engine, and can be used alongside ESIM to generate event data. An external method of generating event data from the frame-based outputs from IsaacSim is required as the application does not presently support event-based sensors (as of version 5.1.0). The application is aimed towards industrial robotics applications and utilises the Universal Scene Description (USD) format to represent simulated environments. Although the focus of IsaacSim is geared towards robotics, it is possible to simulate a wide variety of scenes in the program, including the movement of pedestrians and vehicles in detailed 3D environments. An example of a virtual environment generated using IsaacSim is shown in Figure 10.

This is demonstrated in a study by Kausar et al. [110]. A quadcopter is simulated in a virtual twin of Khalifa University SAN Campus, Abu Dhabi. Event data is simulated from IsaacSim by capturing the output from a fisheye camera fixed to the quadcopter, then comparing pixel intensity between frames to produce spiking data. In the virtual scene, the quadcopter is able to return to its point of origin autonomously, predicting the vector to return to the home location accurately.

#### 4.4.2. CARLA

Other software allows for event data to be exported directly. CARLA [105] is one such simulator that is capable of rendering the events in a virtual urban environment. The project is aimed towards autonomous driving applications and can be scripted to simulate scenarios using code written for a Python processing engine. Along with event vision data, CARLA can also export data from other sources including LIDAR. CARLA is built on the Unreal Engine platform and can simulate highly realistic scenes from the point-of-view of a vehicle in the scene.

**Figure 11:**
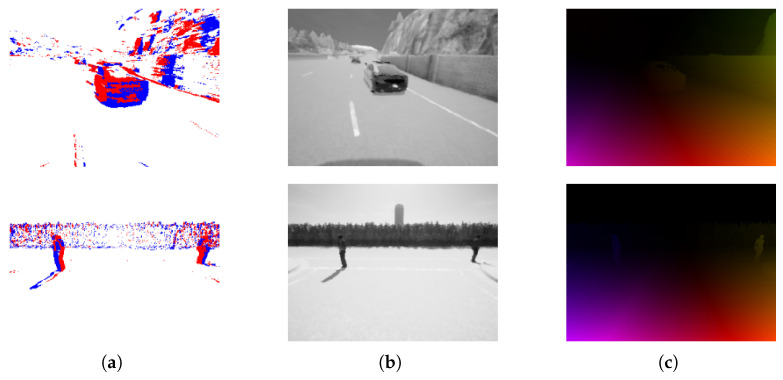
Examples of scenarios represented in the eCARLA-scenes dataset. Frame and event data are generated, along with optical flow where hue and intensity represent the direction and magnitude of relative motion in the scene between frames. Reproduced with permission from Mansour et al., “eCARLA-scenes: A Synthetically Generated Dataset for Event-Based Optical Flow Prediction” [111]; published by arXiv, 2024. (**a**) Event data; (**b**) Frame data; (**c**) Optical flow.

CARLA has been used to generate a dataset of event data representing optical flow in a visual scene, known as eCARLA-scenes [111]. The dataset contains 31 simulated scenes containing pedestrians and vehicles (referred to here as “entities”), where each simulation is approximately 59 s long. The complexity of each simulation increases progressively by introducing more pedestrians and vehicles into the virtual space. Examples of scene generated using CARLA for the eCARLA-scenes dataset are shown in Figure 11.

#### 4.4.3. ANTShapes

**Figure 12:**
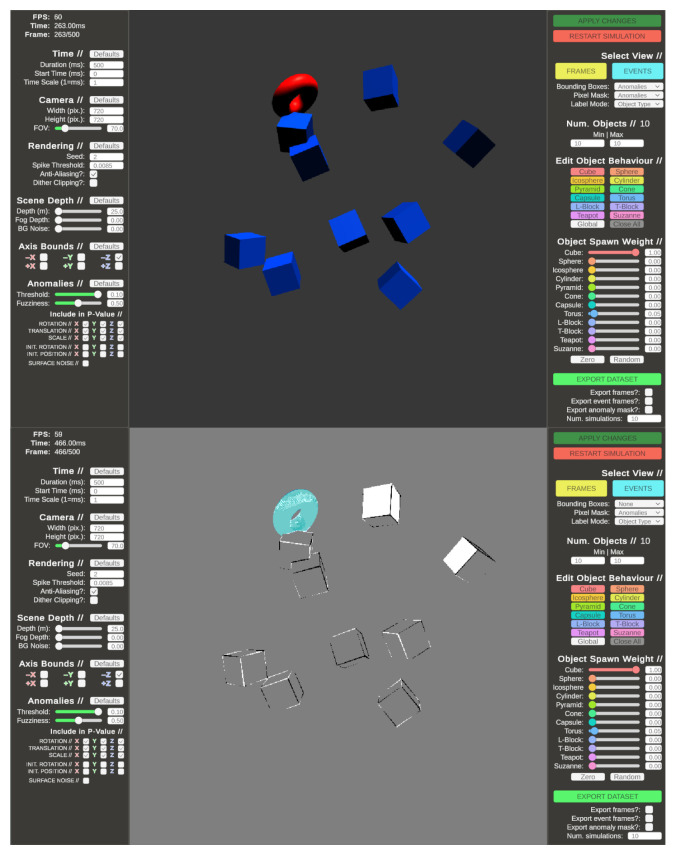
Assorted shapes populating a scene simulated using ANTShapes. **Above**: simulated frame data. **Below**: simulated events. Anomalous entities (toruses) are highlighted by the software.

The Anomalous Neuromorphic Tool for Shapes (ANTShapes) is software developed in the Unity 3D engine to simulate datasets, where anomalous objects are automatically labelled against non-anomalous subjects [106]. The purpose of this software is to model scenes where many individually-acting entities express a reduced pool of common behaviours, such as class of shape, translation and rotation speed. These per-entity behaviours are assigned based on sampling from normal distributions, where the user can specify the desired variance in behaviour among the crowd of objects. Entities can be labelled by behaviour by evaluating how statistically likely it is for them to exist given their behaviours against the mean and variance parameters defined by the user. A scene simulated using ANTShapes is shown in Figure 12.

Similarly to CARLA, ANTShapes allows for the direct export of event-based vision data in an AER-like format. However, whereas scenarios in CARLA must be scripted for simulation, ANTShapes relies on parametrised procedural generation to produce its simulations. Datasets consisting of many unique scenarios can therefore be exported at once, allowing for the fast assessment of SNNs for different tasks using simulated data. For example, ANTShapes can be used to generate datasets for the training of SNNs for object recognition by allowing variance in the classes of objects that can populate the scene, or to simulate object localisation tasks by generating many examples of objects placed in random positions in the scene. Additional effects, such as background noise, camera field of view and distance-based fog can be used to control the signal-to-noise ratio of the generated examples.

The scene simulation model used by ANTShapes is an attempt to model anomalous behaviour in crowds based on a central-limit theorem model. Entities within the scene also do not collide, but rather intersect and pass through each other. This combination of non-physical interaction between entities and the assumed non-causal behaviour in crowds is not analogous to the real world. However, the method of labelling entities as “normal” or “anomalous” based on their likelihood to exist given parametric bounds imposed by the user is statistically valid, despite the issues raised regarding the ability of the tool to model real-world behaviour.

### 4.5. Discussion and Challenges

Given the ubiquity of frame-based datasets for computer vision, it could be more advisable to convert image sequences to spiking data for experimental use, as discussed in Section 4. However, the quality of frame-to-event conversion is largely dependent on the interpolation scheme used to extrapolate from low to high temporal resolution image sequences. The subsequent extraction of events from the interpolated sequence of frames, obtained by comparing the difference in brightness between a pixel at two adjacent timesteps to a bipolar threshold, is relatively trivial. A limitation of frame-to-event conversion is that motion blur present in the source image sequence remains uncorrected. More broadly, visual artefacts represented in the source image sequence will be used for interpolation, and will therefore also result in artefacts in the simulated event stream. Furthermore, if the source frame rate is especially slow, there may not be enough information in the image sequence for reliable interpolation, and the detail of complex motion, such as human gait and pose, will be estimated poorly if captured poorly by the source frame-based sensor.

The availability of software tools to generate event-based datasets purely synthetically is also limited at present. This could be due to the wide-ranging applications of tools such as ESIM, which can be coupled with other 3D modelling software familiar to the user and according to their own workflow. The coupling of industrial scene simulation software such as IsaacSim with ESIM in a pipeline could be used to autonomously produce a rich neuromorphic vision data. At present however, there are no tools available specifically targetted towards whole dataset generation. Researchers must define their own models and paradigms for the datasets they wish to simulate, then create this dataset sample-by-sample. This is beneficial as the user ultimately has more control over the samples included in their dataset, but comes at the cost of time required to produce the dataset.

One key advantage of dataset simulators is the ability to generate behaviour in scenes that is difficult to capture in real-world scenes. For example, a virtual pedestrian could be scripted to step out in front of a vehicle using CARLA, or industrial accidents could be scripted using IsaacSim. Capturing such behaviour in real life poses significant safety and ethical concerns, despite the importance of studying such behaviour in the design of safe autonomous systems. Furthermore, the access to such data for researchers can be restrictive given the sensitive nature of the material, a limitation that is removed by modelling a dangerous scenario virtually.

The challenge for researchers building a simulation is the development of a “digital twin” representing the behaviours desired in the real world. ANTShapes attempts to model the anomalous behaviour that might be displayed by a person in a crowd, but represented in an simplified manner using 3D models of shapes. The central limit theorem-based model of ANTShapes is imperfect at modelling this behaviour, as members in crowds are not expected to follow individual behaviours but adapt the pattern of behaviour from the crowd. Patterns of life, such as crowd behaviour in this example, may require more complex modelling to produce faithful digital twins of real-world scenarios; a suggested approach for modelling crowd behaviour is the social force model. This limitation cannot be addressed purely by improving the visual fidelity or complexity of a simulated scene, as the fundamental issue is related to the scripted behaviour of entities within the virtual scene. A scenario created in CARLA or IsaacSim that does not model the real-world situation that is the target of research efforts can introduce more visual noise into the problem. This is in comparison to an accurate virtual model of the desired behaviour as performed in an abstract representation of the world, such as a simplified representation of the scenery and geometry used for the simulation.

The relative lack of neuromorphic data available to researchers in comparison to framebased video data has been established as a barrier to entry in Section 3. The methods of simulating data presented here are means towards closing this gap. Whilst the conversion of frame to event data may not be ideal, it is a practical alternative in the present for researchers who cannot get a dynamic vision sensor for their purposes. Even if the integration gap in hardware availability is closed in the future, there is a need to continue the development of digital twin simulators to model events that are impractical to capture in real life.

## 5. Neuromorphic Computing Hardware

Neuromorphic computing, or brain-inspired computing, is the engineering challenge of developing compute hardware that mimic certain desirous properties of brain function. Neuromorphic machines are designed to support SNNs and similar workloads, with lower latency and power requirements compared with more general compute architectures. Owing to the breadth and complexity of brain function, the research community tends not to formally define the set of characteristics that make a machine neuromorphic: here, we review computing hardware that self-identifies as neuromorphic, and is able to support SNN workloads effectively.

The development of neuromorphic computing hardware has been historically motivated by two distinct, yet mutually-informed causes. One is the enablement of computational neuroscience modelling of large-scale SNNs with the objective of understanding how the mammalian brain represents and processes information. Research efforts dedicated to this cause have led to massively parallel computing architectures that have, on one hand, assisted neuroscientists in exploring hypotheses about proposed computational brain models and, on the other, enhanced the available toolsets for parallel computation.

Power-efficient machine learning is the other cause which is closely aligned with machine vision applications at the edge. In contrast to non-spiking ANNs which rely on Multiply-and-Accumulate (MAC) operations for both inference and training, the inherent sparsity within spiking data representation has the potential of decreasing the computational load. Therefore, replacing ANNs by SNNs for machine learning tasks can lead to a decrease in the overall energy consumption, provided that processing takes place on hardware that takes advantage of the spiking data representation.

**Figure 13:**
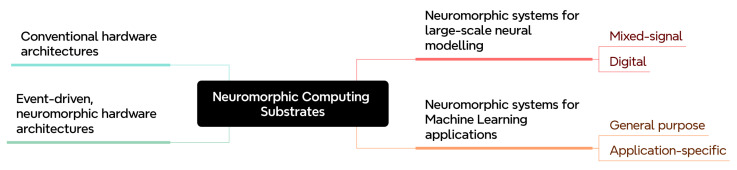
Taxonomy of hardware platforms used for neuromorphic computing.

In this section, we present a snapshot of the spectrum of neuromorphic computing hardware available to date, following the taxonomy presented in Figure 13. We first cover neuromorphic hardware designed with the goal of supporting multiple SNN topologies for neural modelling (Section 5.1). Second, we focus on designs targeted at machine learning applications (Section 5.2). Hardware falling under this category is designed under the constraints of power consumption, die area and degradation of machine learning evaluation benchmarks. Next, we review the role of non-dedicated, conventional hardware architectures in the training and evaluation of SNNs through software frameworks typically used for non-spiking ANNs (Section 5.3). We present event-triggered computing platforms which can support SNN deployment (Section 5.4). Lastly, in Section 5.5 we provide a summary of our observations about the evolution of neuromorphic hardware and discuss suitable candidates for vision applications at the edge.

We present a specification overview of the covered neuromorphic systems in Table 4, demonstrating the increasing integration of neurons and synapses per chip with improvements in fabrication technology. Given the varied nature of the covered systems, this table aims at highlighting key characteristics instead of enabling direct comparisons.

### 5.1. Neuromorphic Hardware Platforms for Neural Modelling

The major point of distinction between neuromorphic hardware systems designed for large-scale neural modelling is the choice of neuron representation: either as a circuit, or as a software module executed on a digital processor. Using digital or mixed-signal electronic circuits that emulate neuron behaviour as the fundamental building block for a neural modelling system has the advantage of decreased power consumption in comparison to using software for the same task. However, software approaches are reprogrammable, and better support initial exploration of ideas, or comparisons of algorithmic approaches.

#### 5.1.1. Mixed-Signal Architectures

The Heidelberg BrainScaleS [112] system developed under the FACETS project was based on mixed-signal VLSI circuits to emulate neuron behaviour. Specifically, the system used the exponential Integrate and Fire (AdExp) neuron model (also developed under the same project), implemented in 180 nm CMOS technology. To reduce the neuron’s internal capacitances, the model, and hence the whole system, was operated at an accelerated time scale, with an acceleration factor of up to 10^5^ compared to Biological Real Time (BRT). Multiple neurons along with their respective synapses were integrated together into Analog Network Cores (ANCs), containing up to 512 neurons. Communication between neurons occurred through an asynchronous, serial event protocol called Layer 1 routing. The final system was built out of 20 interconnected wafers, each containing 352 ANCs.

The BrainScaleS-2 [113,120] architecture was also driven by the goal of enabling largescale neural network emulation at accelerated BRT. As with its predecessor, a total of 512 AdExp neuron circuits were included in each core. However, the synapses were organised into four quadrants of 256 × 128 crossbars instead of two blocks of 224 × 256 arrays. Communication was facilitated by a cross-shaped network located in between the four quadrants. Two digital processors located at the top and bottom of the core were added to read and write the digital state of the synaptic crossbars via SIMD instructions.

The system developed by the Neurogrid project [114] was also based at a mixed-signal architecture. Its main building blocks, called Neurocores, where connected in a binary tree network topology. Each Neurocore consisted of a 256 × 256 silicon-neuron array which was designed with the goal of reducing transistor count by sharing synapses and dendritic tree circuits among neurons. The proposed Shared Dendrite architecture (SD) provided a reduction in the silicon area required per synapse, allowing the integration of a higher number of neurons under the same area constraints. However, there was a consequent increased synapse activation time, which increased latency. On the other hand, the Fully Dedicated architecture (FD) of the BrainScaleS system, where axons and synapses were dedicated to individual neural elements, provided the reverse of this trade-off: area was sacrificed for a decrease in synapse activation time. Consequently, adopting a SD architecture favours modelling applications that require a high number of neurons with mostly local connectivity (i.e., modelling the neocortex), whereas a FD architecture is more suitable for applications that need to run faster than BRT and require arbitrary neural connectivity (i.e., modelling neural development).

#### 5.1.2. Digital Architectures

The SpiNNaker [115] project introduced a massively-parallel architecture based on general-purpose processor cores. Each SpiNNaker chip/node incorporated 18 ARM968 cores equipped with 96 kB of local memory and 128 MB of shared memory. Neuron models were represented by software modules executing within the cores. Transmission of generated spikes occurs through a multicast packet-routing mechanism implemented as an extension of conventional Address Event Representation (AER), which is considered as the project’s key innovation. The SpiNNaker system was assembled by 48-node PCBs. Each node is connected with its six neighbour nodes via a 2D triangular mesh network, forming a torus-shaped network at a system level.

### 5.2. General-Purpose Neuromorphic Hardware for Machine Learning

Neuromorphic hardware developed for machine learning applications can be further segregated into general-purpose designs that support multiple SNN topologies for variety of ML tasks and application-specific designs optimised for a fixed SNN architecture and task. In this subsection, we review the major general-purpose hardware platforms developed by academic institutions and industry.

SpiNNaker2 [116,121] was introduced as a versatile accelerator for event-based and asynchronous computation. In contrast to its predecessor, it was designed as an alternative to conventional hardware accelerators, such as GPUs (Graphics Processing Units) and TPUs (Tensor Processing Units), for machine learning applications, deviating from the design intents of the original SpiNNaker system motivated by computational neuroscience. Despite this shift, SpiNNaker2 retained the foundational architectural elements of the original system. Each stand-alone SpiNNaker2 chip/node contained 152 ARM Cortex M4F cores with 128 kB of local memory and 2 GB of shared, off-chip DRAM. Additional hardware was introduced to each core to allow common operations required by typical ML workloads such as matrix multiplication, 2D convolution and pseudo random number generation. Event communication between cores and nodes was facilitated by the same networking principles as with the original SpiNNaker system.

The IBM TrueNorth chip [117] was the outcome of another digital design effort adopting the principles massively-parallel, event-driven and scalable architectures. Each chip was built out of 4096 neurosynaptic cores connected by a 2D Mesh network. The synapses of each core were organised into a 64 × 64 crossbar array and the neuron equations of the Leaky Integrate and Fire model were followed to produce spikes. While the intra-chip and inter-chip communication between the cores occurs asynchronously, computational circuits operate synchronously following local clock signals.

The Loihi chip [118] was Intel’s first attempt in producing a programmable hardware platform for SNNs targeted to ML and optimisation applications. It featured 128 cores arranged in a 2D mesh and three embedded x86 processors cores. As with other digital architectures discussed so far, cores communicate asynchronously through packetized messages distributed on a Network-on-Chip (NoC). The unicast mesh routing protocol can be scaled up to 4096 on-chip cores for a total of 16,384 chips. Each core implements a total of 1024 Generalized LIF neurons with up to 10^4^ fan-outs through the support of sparse matrix connectivity models, variable synaptic weight precision and weight sharing via populationbased hierarchical connectivity. Each core features a learning engine which can update synaptic weights following learning rules defined by the user through programmable microcode.

Loihi 2 [119] extended the functionality of its predecessor through a number of additions and changes. Some of them include: an increase in the the number of x86 processors responsible for housekeeping operations from three to six per chip, fully programmable neuron models in the place of Generalized LIF neurons, multicast packet routing instead of unicast, replacement of binary spike events by graded spike events with 32-bit payloads and support for 3D multi-chip scaling which reduced inter-chip routing distances.

### 5.3. Conventional Hardware Platforms

Despite the rapid advances in the field of neuromorphic hardware, access to the majority of the platforms discussed in Section 5.2 remains restricted to a small portion of the research community. On the other hand, the widespread success of deep learning has led to increasing adoption of GPUs for research purposes by both academia and industry. As a natural consequence, experimentation with a variety of SNN architectures often occurs through the use of software frameworks optimised for conventional hardware backends. In this section, we review common software frameworks which are popular with the SNN research community.

As with neuromorphic hardware, SNN software frameworks can be separated to packages that were originally targeted to neural modelling for computational neuroscience research or developed for ML applications. Characteristic examples of packages belonging to the first category are the Python-based SNN simulators Brian2 [122] and PyNEST [123]. These packages allow experimentation with biologically plausible neuron models and networks structures but have no built-in support for GPU backends. However, NEST allows CPU parallelism via message passing interfaces, enabling the mapping of large-scale models to multi-core hardware backends.

As far as the second category is concerned, the familiarity of ML researchers with conventional deep learning tools such as TensorFlow [124] and PyTorch [125] has motivated the neuromorphic community to augment the functionality of existing frameworks to support SNNs. Popular efforts dedicated to this cause are the PyTorch-based packages snnTorch [126], Norse [127], SpikingJelly [128] and BindsNET [129] which allow SNN training via surrogate-gradient descent [130]. Aiming to bridge the gap between conventional and neuromorphic hardware, Intel introduced the LAVA [131] framework as a platformagnostic software package for the development of neuro-inspired applications. LAVA allows users to define SNN topologies via a Python-based interfaces which is compiled for the target backend via LAVA’s low-level interface, called Magma. Despite LAVA’s open-source nature, the toolchain for the Loihi backend remains proprietary, which poses a barrier for the research community.

To address the need for interoperability between different SNN software frameworks, the Neuromorphic Intermediate Representation (NIR) [132] was proposed as a common set of computational primitives that can be used to define computational graphs for SNNs. Serving a similar cause as the Open Neural Network Exchange (ONNX) [133] representation used by the deep learning community, NIR provides a framework-agnostic description for an SNN architecture which can be generated/imported by frameworks with NIR support.

### 5.4. Event-Driven Neuromorphic-like Hardware Platforms

Developments in neuromorphic computing hardware have also resulted in effective progress in the development of hardware platforms with certain neuromorphic characteristics: neuromorphic computing hardware and algorithms have demonstrated the efficacy of computing in response to events. By entering a state of dormancy, and by waking up only when computation is timely, networks of neurons (and more generally, networks of compute nodes) save considerable power while achieving an incredible degree of parallelism. It is natural to adapt these properties outside the neuromorphic computing space for more general computation, on hardware that is more general in purpose.

The POETS project is an exemplar that attempted this by implementing a bespoke manycore architecture across a network of FPGAs, alongside a software toolchain for describing and running massively-parallel compute problems [134,135]. While the architecture is bespoke, the goal of POETS was to explore a general set of non-neuromorphic compute problems that are massively-parallel in nature, but that also have great communication requirements. What resulted was orders-of-magnitude performance improvements across a variety of domains, including computational chemistry [136], magnetic simulation [137], genomic imputation [138], and graph analysis [139].

Ultimately, POETS (and similar “neuromorphic-like” architectures) scale effectively on parallel compute problems for which the communication is a significant bottleneck to performance on more traditional high-performance computing platforms. The challenge primarily lies in the expression of problems in an event-driven format, which typically requires a reformulation of the problem specification, and significant development of bespoke algorithms. In learning from the way the brain performs computation, more effective parallel computing approaches have developed, and will continue to develop as neuromorphic computing, as a field, matures.

### 5.5. Discussion and Challenges

The review of hardware platforms developed and adopted over the years for experimentation with SNNs reveals a number of trends. While earlier neuromorhpic systems such as Neurogrid, BrainScaleS and SpiNNaker were built with the goal of enabling large-scale neural modelling, the success of deep learning has shifted the focus to the design of systems with ML applications in mind. This transition has been clearly demonstrated by the design of SpiNNaker2, which incorporated hardware accelerators particularly targeted to operations used frequently for deep learning workloads, while preserving the key architectural elements of its predecessor.

The second trend that we observe is a gradual abandonment of mixed-signal architectures and a stronger focus on digital architectures that emphasize neuron model configurability and SNN architectural flexibility. Mixed-signal architectures have demonstrated the benefits of higher integration density, allowing higher numbers of neurons and synapses per core when compared to their digital counterparts, as well as accelerated BRT operation. However, the improvements in fabrication technology and involvement of industry into neuromorphic hardware development have led to highly configurable digital architectures with higher degrees of neural integration.

We consider the adaptation of software frameworks, originally developed for ANN training on conventional hardware, for SNNs as another important trend. Its major benefit is the enablement of rapid experimentation with SNN architectures on hardware that is readily available to the majority of neuromorphic researchers.

Despite the advances in maturity of general-purpose neuromorphic hardware for ML applications, we observe two challenges that prevent its adoption for research within the domain of event-based vision at the edge.

The first one is the lack of off-the-shelf development kits. In contrast to event-based sensors such as DVS cameras, where there is already a market in place, access to generalpurpose neuromorphic chips for research projects is facilitated only via special agreements formed between research institutions and industry. An effort to facilitate such collaborations has been the introduction of Intel Neuromorphic Research Community (INRC), through which either remote or physical access to Loihi chips may be provided. However, there is still a substantial distance too be covered towards making such hardware widely available.

The second challenge is the limited software support for neuromorphic hardware backends suitable for edge applications. The growing adoption of NIR representation by SNN software frameworks aimed at conventional hardware architectures decreases the effort required to port models to frameworks that support neuromorphic hardware. However, as discussed in Section 5.3 support for neuromorphic backends is enabled via proprietary toolchains (i.e., LAVA’s Magma low-level interface). Lack of access to such toolchains prevents the characterization of key metrics such as inference power and latency, prohibiting direct comparisons between event-based and frame-based vision approaches.

A third challenge concerns the physical constraints imposed by edge deployment environments. Neuromorphic hardware platforms designed for large-scale neural modelling, such as SpiNNaker, are not suitable for deployment at the edge owing to their size and power draw. Alternative small-scale platforms, such as Loihi 2, are more suited for deployment at the edge. For example, a complete event-based inference pipeline integrating the Sony IMX636 event camera with a Loihi 2 chip has been demonstrated to consume just 90 mW in a fall detection application [140].

Recent benchmarks for sensor fusion workloads demonstrate that Loihi 2 can achieve energy efficiency over 100 times greater than a CPU and approximately 30 times greater than a GPU [141]. However, these figures reflect chip-level measurements for individual workloads rather than complete system pipelines. The size, weight, and power envelope of a full inference pipeline is rarely reported as a unified metric in the literature. This is compounded by restrictions on the access to neuromorphic hardware; without physical devices, SWaP budgets cannot be empirically validated, and the efficiency gains suggested by chip-level benchmarks are difficult to translate into system-level conclusions for edgetargeted vision applications.

From this discussion, gaps in integration of neuromorphic hardware into an eventbased vision system can be identified. Similarly to neuromorphic vision sensors discussed in Section 3, the availability of neuromorphic computing hardware relative to conventional computing hardware restricts their use in a computing pipeline for research or deployment. This makes the investigation of power efficiency difficult; if no hardware is available, estimations on potential reductions in power consumption must be estimated. In addition, the requirement to reach an agreement with an industry partner is a far greater barrier to access than cost alone. The literature available surrounding neuromorphic computing is far less mature than conventional computing, creating an additional barrier that can only be overcame by continued research, and by allowing researchers improved access to hardware through additional manufacture or looser terms to purchase neuromorphic chips. In contrast, the availability of packages such as snnTorch allow for research into the dynamics of neuromorphic computing systems without the need for the computing hardware. Whilst this closes the gap between integration of neuromorphic hardware into a computer vision pipeline, the mappings between toolchains and neuromorphic hardware are not exact. For example, a neural network built using snnTorch may run differently to an equivalent network hosted on Loihi 2 if restrictions on fixed-point computations are not replicated carefully in the snnTorch simulation. The recent introduction of NeuroBench [142], a community-designed framework providing comparisons of neuromorphic models, may address this by allowing for fair and objective comparisons to be made between modelling approaches.

## 6. Conclusions

The availability of event-based vision data for the training of neuromorphic systems continues to be a challenge given the relative difficulty in obtaining a dynamic vision sensor in comparison to a frame-based video sensor. For this reason, the simulation of events through digital means will continue to be a point of interest until the issues with demand for event-based sensors can be addressed.

Neuromorphic hardware has evolved from neuroscience-driven, mixed-signal systems toward highly configurable digital platforms optimised for machine learning, reflecting both advances in semiconductor technology and the increasing involvement from the industry. At the same time, the reliance of the SNN research community on conventional hardware has grown significantly, a trend which have been propelled by the adaptation of common deep-learning frameworks to enable accessible experimentation with SNN architectures. Despite these advances, adoption for event-based vision applications at the edge remains constrained by limited neuromorphic hardware availability and restricted software toolchains, which hinder broad access, reproducible evaluation, and direct comparisons with conventional, frame-based approaches.

Several barriers to integration of neuromorphic vision systems into practical computer vision systems have been discussed in this paper. Those relating to the pace and direction of research, such as optimal SNN architecture and training methods, will naturally be addressed as the field matures. The challenge is in allowing the field of neuromorphic computing to develop quickly. In practical terms, this means improving the access to neuromorphic sensors and computing platforms by lowering the cost and contractual barriers to entry. However, there is a feedback effect; an increase in research interest motivates the increased production of hardware, in turn improving accessibility and attracting further research. To keep this feedback loop active, the results demonstrated by SNN algorithms must continue to be promising.

The review presented in this paper demonstrates that the field of neuromorphic computer vision is in its developing stages, and that there are many challenges to be overcome before neuromorphic systems can be meaningfully deployed at the edge. Researchers of neuromorphic computer vision face fundamental challenges at every stage; from data acquisition to processing and interpretation. We believe that the continued collaboration between DVS manufacturers, data scientists, neuromorphic computing engineers and industrial partners will result in these problems being addressed as the field matures.

## Figures and Tables

**Table 1:** Recent state-of-the-art spiking CNNs and Transformers on ImageNet-1K. *T* = simulation timesteps. **Bold** = best in group. “–” = not reported.

Model	Training	*T*	Params (M)	Top-1 Acc. (%)
*CNN-based Spiking Neural Networks*				
SEW-ResNet-152 [1]	Direct	4	60.2	69.26
MS-ResNet [22]	Direct	4	–	**74.21**
*Transformer-based Spiking Neural Networks (Direct Training)*				
Spike-Driven Transformer [23]	Direct	4	–	77.10
SpikingResformer [24]	Direct	4	–	79.40
SNN-ViT-8-512 [25]	Direct	4	53.7	80.23
Spikformer V2 [26]	Direct	4	–	80.38
Meta-SpikeFormer [27]	Direct	4	–	80.50
SGLFormer [28]	Direct	4	64.0	**83.73**
*ANN-to-SNN Conversion*				
QCFS ResNet-34 [29]	Conversion	64	21.8	72.35
Dual-Threshold ResNet-34 [30]	Conversion	64	21.8	73.31
SViT-L-32Level [31]	Conversion	64	304.3	**83.82**

**Table 2:** Details on datasets discussed in this section.

Dataset	Year 1st Pub.	Purpose	Spatial Resolution	Total Examples
N-MNIST [72]	2015	Multi-class classification	34 × 34	70,000
MNIST-DVS [73]	2015	Multi-class classification	128 × 128	30,000
N-Caltech101 [72]	2015	Multi-class classification	240 × 180	8246
N-Cars [74]	2018	Binary classification	304 × 204	24,029
MVSEC [75]	2018	Complex scene	346 × 260	53
DVSGesture [76]	2017	Multi-class classification	304 × 204	1342
E-VLC [77]	2025	Complex scene	1280 × 720 (events), 1280 × 1024 (frames)	146,000 (total frames)

**Table 3:** Summary of data simulation tools discussed in this section that directly export event-based data.

Method/Tool	Year 1st Pub.	Purpose	Primary Approach	Visual Noise Model
ESIM [99]	2018	Event extraction and 3D rendering	Temporal sampling with frame interpolation	None
DVS-Voltmeter [100]	2022	Noise-aware frame-to-event conversion	Model of DVS circuit behaviour	Brownian motion
Raw2Event [101]	2025	Real-time conversion on standard hardware	Estimation from raw Bayer frames	Circuit modelling
EventGAN [102]	2019	Adversarial event estimation	GAN-based model	Learned by GAN
v2e [103]	2021	Video-to-event conversion toolbox	Super SloMo interpolation	Fluctuating thresholds
v2ce [104]	2024	Continuous event prediction	U-net model	Density map estimation
CARLA [105]	2017	Autonomous driving simulation	Unreal Engine simulation	Realistic rendering
ANTShapes [106]	2026	Anomaly detection	Unity 3D Simulation	Gaussian

**Table 4:** Specifications of neuromorphic hardware covered in this section.

System	Year 1st Pub.	Domain	Interconnect	Neurons/Core	Synapses/Core	Cores/Chip	Area	Techn. Node (nm)
BrainScaleS [112]	2010	Mixed-signal	Async. Serial Bus	512	110 × 10^3^	352 (wafer)	20 cm^2^ (wafer)	180
BrainScaleS-2 [113]	2022	Mixed-signal	Cross-shaped AER Bus	512	131 × 10^3^	1	32 mm^2^	65
Neurogrid [114]	2014	Mixed-signal	Binary Tree Multicast	65 × 10^3^	10^6^	1	168 mm^2^	180
SpiNNaker [115]	2009	Digital	2D Torus Multicast	10^2^	10^5^	18	102 mm^2^	130
SpiNNaker2 [116]	2018	Digital	2D Torus Multicast	10^3^	10^6^	152	103 mm^2^	22
TrueNorth [117]	2015	Digital	2D Mesh Unicast	256	65 × 10^3^	4096	430 mm^2^	28
Loihi [118]	2018	Digital	2D Mesh Unicast	1024	10^6^	128	60 mm^2^	14
Loihi 2 [119]	2021	Digital	2D Mesh Multicast	8192	10^6^	128	31 mm^2^	7

## Data Availability

No new data were created or analyzed in this study.
